# Potential link between the RagA-mTOR-p70S6K axis and depressive-behaviors during bacterial liposaccharide challenge

**DOI:** 10.1186/s12974-019-1610-5

**Published:** 2019-11-11

**Authors:** Jia Zhao, Lixing Lao, Wei Cui, Jianhui Rong

**Affiliations:** 1 School of Chinese Medicine, Li Ka Shing Faculty of Medicine, The University of Hong Kong, 10 Sassoon Road, Pokfulam, Hongkong China; 2 Department of Chinese Medicine, The University of Hong Kong Shenzhen Hospital, Shenzhen, China; 3 Hong Kong Branch of Zhu’s School of Gynecology of Chinese Medicine from Shanghai Workstation of Zhu Nansun, National Master of Chinese Medicine, Hong Kong, China; 4 Yu Jin, Master of Gynaecology of Chinese Medicine and Integrative Medicine, Integrative Medicine Workstation for Training and Research (Hong Kong Branch), Hong Kong, China; 5 Ningbo Key Laboratory of Behavioral Neuroscience, Zhejiang Provincial Key Laboratory of Pathophysiology, School of Medicine, Ningbo University, Ningbo, China; 6 The University of Hong Kong Shenzhen Institute of Research and Innovation (HKU-SIRI), Shenzhen, China

**Keywords:** Depression, LPS, RagA, mTOR, p70S6K

## Abstract

**Background:**

Bacterial infection is a potential risk factor for depression. However, little is known about the mechanistic link between bacterial endotoxin and depressive-like behaviors. The aim of the present study was to clarify whether liposaccharide (LPS) could induce depressive-like behaviors in mice via sequentially activating small GTPase RagA, mammalian target of rapamycin (mTOR), and p70S6K.

**Methods:**

C57BL/6 N mice were treated with 0.83 mg/kg LPS by intraperitoneal injection for 24 h. The animals were assessed for depressive-like behaviors by forced swim test and tail suspension test. The expression levels of RagA, mTOR, and p70S6K were determined in mice, primary cortical neurons, neural stem cells, and PC12 cells.

**Results:**

LPS effectively induced depressive-like behaviors in mice. Biochemical examination revealed that LPS not only upregulated RagA expression but also activated mTOR/p70S6K pathway in mouse brains. LPS challenge also achieved a similar effect in primary cortical neurons, neural stem cells, and PC12 cells. Following the silencing of RagA expression with specific siRNA, LPS failed to induce mTORC1 translocation to the lysosomal membranes in PC12 cells. These results suggested that LPS might sequentially upregulate RagA and activate mTOR and p70S6K pathways in mice and neural stem cells.

**Conclusions:**

This study for the first time demonstrated that LPS might induce depressive-like behaviors in mice via the upregulation of RagA and subsequent activation of mTOR/p70S6K pathway. Such information may highlight the RagA-mTOR-p70S6K signaling cascade as a novel therapeutic target for the development of new anti-depressant therapeutics.

## Introduction

Depression has recently emerged as a major healthcare issue for affecting 13–20% of the global population [1]. Epidemiological studies suggest that depression not only affects the quality of daily activities but also increases the risk of disability and suicide in patients [2, 3]. Among much effort to understand depression over the past many years, Duman et al. found that depression caused the neuronal atrophy and reduced the synaptic connections in the cortex [4]. Depression might be an inflammatory disease [5]. Endotoxin lipopolysaccharide (LPS) could induce acute inflammatory reactions and cause depressive-like behaviors in mice [6–8]. As a result, LPS was widely used to induce depression in animal models for preclinical studies. Thus, it is a pressing need to elucidate the mechanisms underlying LPS-induced depressive behaviors [9–11].

Mammalian target of rapamycin (mTOR) not only regulates cell growth and metabolism, but also orchestrates the neuronal signaling and excitability [12]. Several previous studies demonstrated that LPS could induce depressive behaviors. These studies also revealed that LPS could activate mTOR signaling pathway [13, 14]. Interestingly, other studies reported that some antidepressant drugs ameliorated depression via targeting the mTOR pathway [15–17]. Protein mTOR forms two different multi-component complexes, mTOR complex 1 (mTORC1), and mTOR complex 2 (mTORC2), respectively [18]. The mTORC1 signaling pathway is known to regulate cell growth, cell proliferation, protein synthesis, and transcription [19]. Dysregulation of the mTORC1 signaling pathway is implicated in the pathology of cancers, diabetes, and amyotrophic lateral sclerosis [19, 20]. Amino acids promote mTORC1 to translocate to the lysosomal surface and form complex with Rag GTPases, leading to mTORC1 activation [20, 21]. Rag GTPases exist in four isoforms: RagA, RagB, RagC, and RagD. Different isoforms form heterodimer (e.g., RagA/RagB, RagC/RagD) to facilitate the deposition and activation of mTORC1 at the lysosomal membrane [22, 23]. Upon activation, mTOR forms protein complex with raptor and is subsequently translocated to the lysosome membrane [24]. Lysosome-associated membrane protein (LAMP2) is a well-characterized lysosomal marker so that the lysosomes are widely visualized by immunostaining with LAMP2 antibody in the cells or tissues [25]. When lysosomes were damaged, LAMP2 was preferentially ubiquitinated for proteasomal degradation [26]. After the deposition to lysosomal membrane, mTORC1 activates several downstream protein targets including p70 ribosomal S6 kinase (p70S6K) and eukaryotic translation initiation factor 4E-binding protein 1 (4EBP1) [15]. A recent study demonstrated that the aberrant activation of mTORC1/p70S6K pathway caused depressive behaviors [27]. However, little is known about the link between mTORC1 activation and LPS-induced depressive-like behaviors.

Our pilot experiments suggested that LPS upregulated the expression of RagA mRNA in mice. The aim of the present study was to test the hypothesis that LPS might induce depressive-like behaviors in mice via activating RagA, mTOR, and p70S6K pathways. We induced depressive-like behaviors in C57BL/6 N mice with bacterial LPS. We assessed depressive-like behaviors and analyzed the expression of signaling molecules (e.g., RagA, mTOR, and p70S6K) in mice, neural stem cells, and primary cortical neurons.

## Materials and methods

### Antibodies and biochemical reagents

Antibodies against p70S6K, phospho-p70S6K, mTOR, phospho-mTOR, RagA, and GAPDH were purchased from Cell Signaling Technology (Boston, MA, USA). mTOR antibody was purchased from Thermo Fisher Scientific (Waltham, MA, USA). The anti-rabbit HRP-conjugated IgG secondary antibody was purchased from Sigma–Aldrich (St. Louis, MO, USA). Lysosome-associated membrane protein 2 (LAMP2) and NeuN antibodies were obtained from Abcam (Cambridge, MA, USA). Alexa Fluor 594-conjugated goat anti-rabbit IgG, Alexa Fluor 488-conjugated goat anti-mouse IgG secondary antibody, and lipofectamine RNAiMAX Transfection Reagent were purchased from Invitrogen (Carlsbad, CA, USA). Protein Assay Dye Reagent Concentrate was purchased from Bio-Rad (Hercules, CA, USA). Select chemiluminescence (ECL) detection kit was purchased from GE Healthcare (Uppsala, Sweden). Dulbecco’s modified Eagle’s medium (DMEM)/F12, DMEM, Opti-MEM reduced serum medium, horse serum, fetal bovine serum, NeuroBasal medium, N2, B27, penicillin and streptomycin, epidermal growth factor, and basic fibroblast growth factor were purchased from Thermo Fisher Scientific (Waltham, MA, USA). On-target plus SMARTpool siRNA for RagA and On-target plus Non-targeting siRNA were purchased from Dharmacon (Lafayette, CO, USA). LPS, temsirolimus, rapamycin, Hoechst 33342, propidium iodide (PI), Accumax solution, Poly-D-lysine, and other biochemical reagents unless otherwise indicated were purchased from Sigma–Aldrich (St. Louis, MO, USA).

### Animals and drug administration

Adult male C57BL/6N mice (7 weeks, 19–23 g) were supplied by the Laboratory Animal Unit, University of Hong Kong. All experimental procedures were performed in compliance with the guidelines of the Committee on the Use of Live Animals in Teaching and Research of the University of Hong Kong (CULATR 3872-16). Animals were housed in a temperature- and humidity-controlled environment on a 12-h light/dark cycle and allowed free access to standard laboratory mice chow and drinking water. For behavioral tests, animals were individually tested one at a time to avoid carry-over effects. Mice from six different litters were used to minimize litter effects. Behavioral tests were performed between the hours of 09:00 and 15:00 on a 06:00 and 18:00 on/off light cycle to control circadian variations. The drug was dosed as described by the previous experiments [8]. Mice received intraperitoneal (IP) injection of LPS (0.83 mg/kg) for 24 h, while control animals received the same volume of saline. The animal behaviors were assessed after 24 h treatment with LPS or saline.

### Behavioral tests

The depressive behaviors were assessed by forced swim test (FST) and tail suspension test (TST) as previously described [28–30].

#### FST procedure

Briefly, mice were individually placed and forced to swim in an acrylic plastic cylinder (height, 30 cm; diameter, 20 cm) filled with 15 cm of water, conditioned at 23 ± 2 °C. After a 2-min habituation for mice, a blinded observer recorded the time when mice remained floating passively or immobile in the water for a period of 4 min.

#### TST procedure

Mice were hanged up by the tails with adhesive tape to the suspension bar (1 cm height, 1 cm width, 60 cm length), leaving a gap of 20–25 cm between the mouse nose and apparatus floor. Adhesive tapes were applied to the tail at the position of 2–3 mm to the very end. A blinded observer monitored the tests for 6 min and recorded the immobility time with a stopwatch. The mice were considered immobile only when the animals hanged passively and were completely motionless.

### Primary culture of rat cortical neurons

Primary rat cortical neurons were isolated from Sprague–Dawley (SD) rat embryos on embryonic day 17 as described [29]. Briefly, the cortical neurons were carefully dissociated and seeded onto six-well plates at a density of 5 × 10^5^ cells/ml and cultured in NeuroBasal medium containing 2% B27 supplement for 7 days prior to the treatment.

### Cell culture of mouse neural stem cells

Neural stem cells were isolated from the cortex of C57BL/6 N mouse embryos on embryonic day 14 (E14) [31, 32]. The cortex tissues were rapidly dissected and triturated up and down with a pipetman to make a single-cell suspension. Cells were then plated at a density of 1–2× 10^5^ cells/ml in a 90-mm dish and cultured at 37°C under a humidified atmosphere of 5% CO2 and 95% air for 5–7 days. The cells were cultured in the DMEM/F12 supplemented with 1% N2 and 2% B27, 1% penicillin and streptomycin, 20 ng/ml epidermal growth factor, and 20 ng/ml basic fibroblast growth factor. The primary neural stem cell neurospheres were propagated every 2–3 days based on the speed of the cellular growth. The single-cell suspension was made with Accumax solution. For the drug treatments, the single cells were seeded at the density of 0.3–1× 10^5^ cells/ml on the poly-D-lysine-coated 6-well plates for 24 h and treated with LPS for 24 h.

### PC12 cell culture and drug treatments

Rat pheochromocytoma cell line PC12 was purchased from American Type Culture Collection (Manassas, VA, USA) and cultured in DMEM supplemented with 10% heat-inactivated horse serum (HS), 5% heat-inactivated fetal bovine serum (FBS), and 1% penicillin/streptomycin on collagen I-coated dishes at 37 °C under a humidified atmosphere of 5% CO_2_ and 95% air. For drug treatments, the cells were seeded at the density of 1 × 10^5^ cells/ml in 6-well plate for 24 h and then treated with LPS for 24 h.

### Hoechst 33342 and PI staining

Hoechst 33342 was used to stain the entire cell monolayers, whereas propidium iodide (PI) was used to stain necrotic cells as previously described [33, 34]. Briefly, the freshly isolated primary cortical neurons were seeded at a density of 5 × 10^5^/ml and cultured in complete growth medium for 7 days. The neurons were treated with LPS for 24 h. Following staining with 5 μM Hoechst 33342 and 5 μM PI, Hoechst- and PI-positive cells were separately counted under a Zeiss fluorescence microscope (Carl Zeiss, Jena, Germany).

### Western blot analysis

Following drug treatment, the olfactory bulbs were removed and the first 3 mm thick of the entire cortex was subsequently recovered as mouse forebrain cortex for further analyses. The cortical tissues were sliced into small pieces. Cortical tissues, PC12 cells or primary neurons were lysed in ice-cold RIPA buffer containing 150 mM NaCl, 1.0% IGEPAL® CA 630, 0.5% sodium deoxycholate, 0.1% SDS, and 50 mM Tris (pH 8.0) with sonication. The proteins were recovered by centrifugation at 13,000 rpm for 15 min at 4 °C. The protein concentrations were determined with protein assay dye reagent from Bio-Rad (Hercules, CA, USA). The brain proteins (70 μg) and cellular proteins (30 or 50 μg) were resolved by gel electrophoresis on 10% SDS-polyacrylamide gels and subsequently transferred onto PVDF membranes. Following overnight blocking with 5% BSA in Tris-buffered saline containing 0.1% Tween-20 (TBS-T) buffer at 4 °C, the blots were probed with specific primary antibodies, detected with HRP-conjugated secondary antibody, and finally visualized with Amersham™ ECL™ Select western blotting detection reagent from GE Healthcare (Uppsala, Sweden) [29].

### Immunostaining

At the end of 24 h treatment with LPS or saline, the mice were anesthetized with a ketamine/xylazine mixture solution and perfused with 4% paraformaldehyde in 0.01 M phosphate-buffered saline (PBS) (pH 7.4). Mouse brains were recovered, further fixed in 4% paraformaldehyde for 3 days at 4 °C, and then incubated in 30% sucrose in PBS overnight at 4 °C. Following placed in − 80 °C freezer for 24 h, the brain tissues were cut into serial coronal sections with a thickness of ∼ 40 μm. The first 3 mm specimen of the entire cortex were blocked with 5% normal goat serum and 0.3% Triton X-100 in PBS for 2 h at room temperature and then incubated with RagA and NeuN antibodies overnight at 4 °C. After the removal of excessive antibodies, the brain sections were detected with Alexa Fluor 594 conjugated goat anti-rabbit IgG secondary antibody and Alexa Fluor 488-conjugated goat anti-mouse IgG secondary antibody. The cell nuclei were subsequently stained with 4′-6-diamidino-2-phenylindole (DAPI) for 10 min. The immunofluorescence images were acquired on a Carl Zeiss LSM 800 fluorescence microscope (Carl Zeiss Jena, Germany). The fluorescence intensity was measured using NIH ImageJ software (http://imagej.net/ImageJ2) [35].

RagA, mTOR, and LAMP2 were detected by immunostaining as previously described [29]. Briefly, neural stem cells were cultured on poly-D-lysine-coated or collagen I-coated 6-well plates in a complete growth medium. At the end of 24 h treatment with LPS, cells were washed twice with PBS and fixed with paraformaldehyde (3.7%). After the removal of excessive paraformaldehyde, the fixed cells were permeabilized with 0.5% Triton X-100 for 30 min at room temperature and blocked in 5% normal goat serum in PBS for 2 h at room temperature. The cells were probed with antibodies against RagA, mTOR, and LAMP2 overnight at 4 °C cold room. The bound antibodies were detected by Alexa Fluor 594 goat anti-rabbit IgG and Alexa Fluor 488 goat anti-mouse IgG secondary antibodies for 2 h at room temperature. At the end of incubation with antibodies, the cell nuclei were stained with DAPI for 10 min. The immunofluorescent images were acquired on fluorescence microscopy (Carl Zeiss, Jena, Germany).

### siRNA-silencing of RagA expression

To transiently silence RagA expression, PC12 cells were transfected with RagA-specific siRNAs and negative control siRNAs as previously described [36]. Briefly, PC12 cells were seeded on collagen I-coated 6-well plates and then transfected with siRNAs using lipofectamine RNAiMAX Transfection Reagent according to the manufacturer’s instructions. After 72 h of post-transfection incubation, the cells were treated with LPS for 24 h, and the proteins of mTOR and LAMP2 were subsequently detected by immunostaining as previously described [29]. The RagA expression was verified by western blot analysis as described above.

## Results

### LPS induced the depressive-like behaviors in mice

**Fig. 1:**
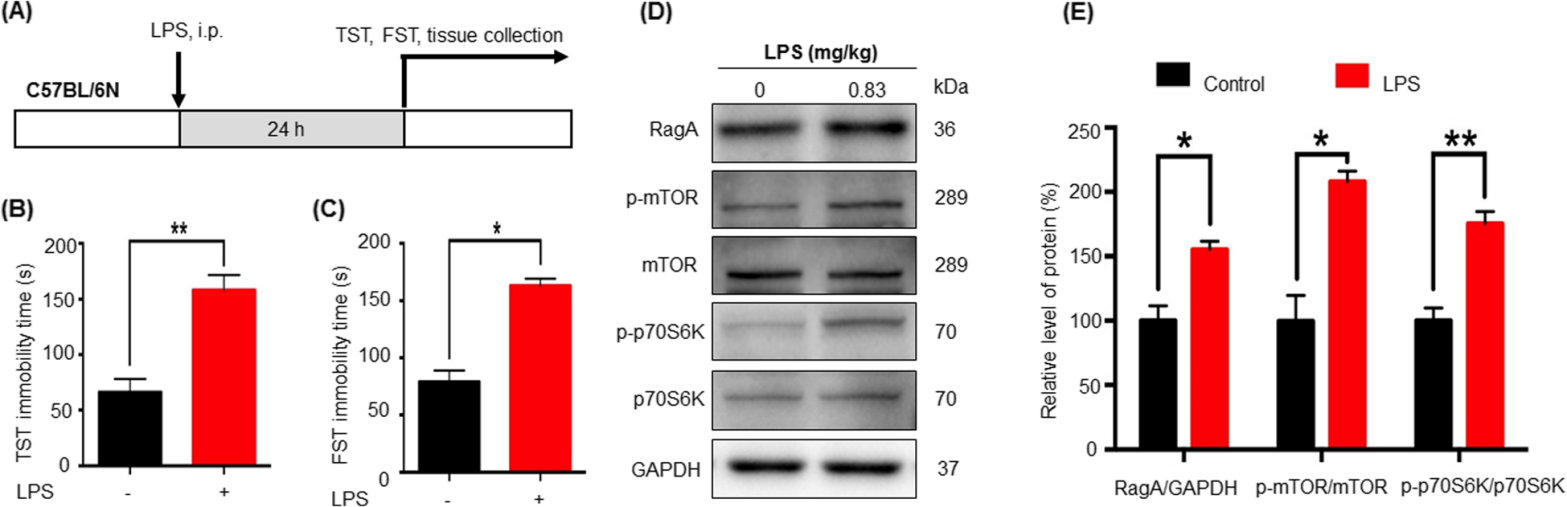
Association of depressive behaviors and RagA-mTOR-p70S6K pathway in LPS challenged mice. **a** Experimental design. Mice were randomly divided into two groups. Mice in the LPS group received single dose of LPS (0.83 mg/kg) by IP injection while control mice received the same volume of saline at 24 h prior to behavioral assessments and tissue collection. **b** TST evaluation. The TST assessment measured the immobility time over a period of 6 min. **c** FST evaluation. The FST assessment measured the immobility time over a period of 4 min. The data were presented as means ± SEM (*n* = 6), and subsequently analyzed by a two-tailed Student’s *t* test. *p < 0.05; **p < 0.01. **d** Western blot analysis of selected proteins. After 24 h LPS treatment, brain tissues were collected and analyzed by western blotting for RagA, mTOR, phospho-mTOR, p70S6K, and phospho-p70S6K expression, whereas GAPDH was analyzed as control. Representative blot was shown. **e** Quantitative analysis for protein expression. Western blots in **d** were determined by a densitometric method. The signals from three independent experiments were presented as means ± SD and analyzed by a two-tailed Student’s *t* test. *p < 0.05; **p < 0.01 (LPS vs control)

To validate the induction of depressive-like behaviors by LPS, we treated C57BL/6N mice with single dose of LPS for 24 h. The animals were evaluated for depressive behaviors by FST and TST protocols as previously described [7]. As shown in Fig. 1b, c, both FST and TST experiments showed that IP injection of 0.83 mg/kg LPS significantly prolonged the immobility time in mice compared with the control animals.

### LPS induced the activation of RagA, mTOR, and p70S6K pathways

**Fig. 2:**
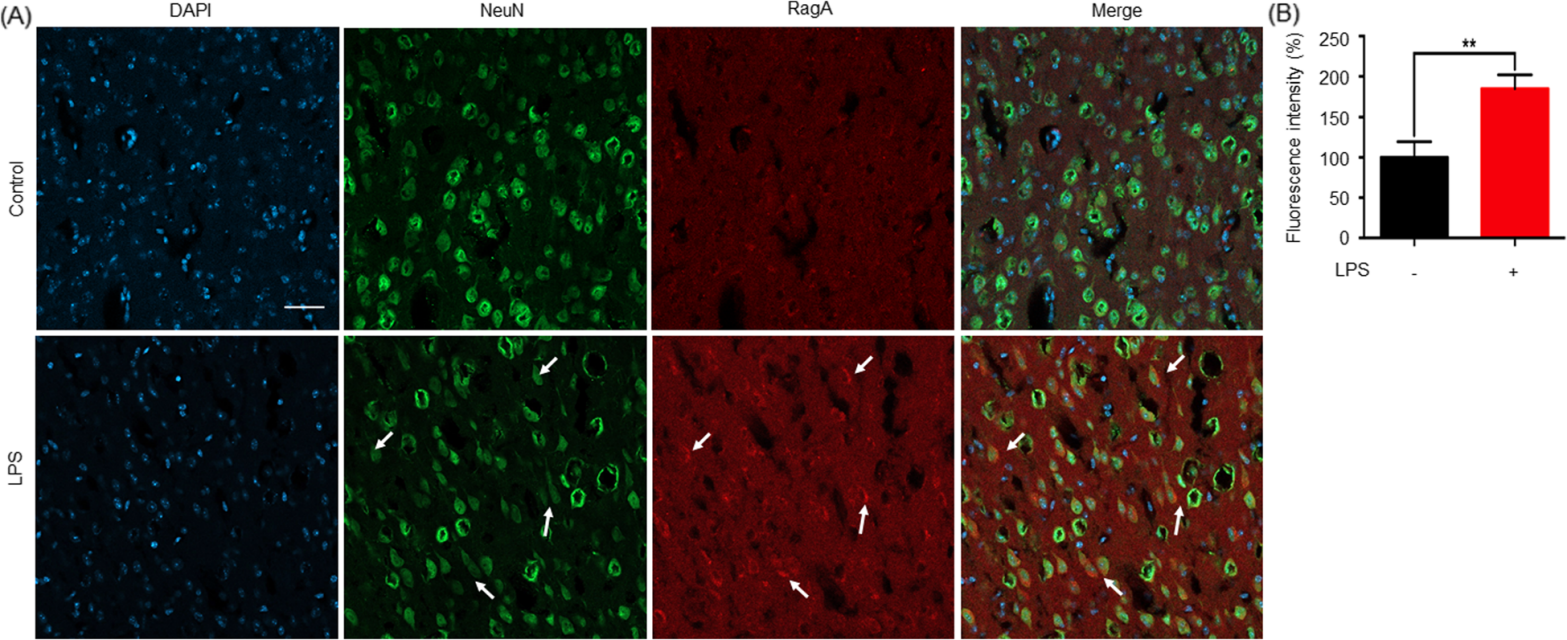
LPS stimulated the expression of RagA in neurons. **a** After 24 h LPS treatment, brain tissues were prepared and analyzed by immunostaining with antibodies against RagA and NeuN while the cell nuclei were indicated by DAPI staining. The stained tissues were imaged on a Zeiss confocal 800 fluorescence microscope (Carl Zeiss, Jena, Germany). **b** Quantitative analysis of RagA levels. Fluorescence intensity of RagA signals in the forebrain cortex was quantified by a densitometric method and expressed in mean ± SD (*n* = 3). The difference between the experimental groups was analyzed by a two-tailed Student’s *t* test. **p < 0.01 (LPS vs control)

To examine the levels of RagA, mTOR, and p70S6K signaling molecules, the forebrain cortex tissues were prepared from mice with or without exposure to LPS. Brain proteins were analyzed by western blotting with specific antibodies. As shown in Fig. 1d, e, LPS significantly increased the levels of RagA, phospho-mTOR, and phospho-p70S6K in mice (*p* < 0.05 and *p* < 0.01, respectively). Moreover, we detected the expression of RagA in the forebrain cortex by immunofluorescence staining. RagA appeared to be co-localized with neuronal biomarker NeuN in the forebrain cortex from LPS-treated mice (Fig. 2a, b).

### LPS enhanced the expression of RagA and phospho-mTOR in the primary cortical neurons

**Fig. 3:**
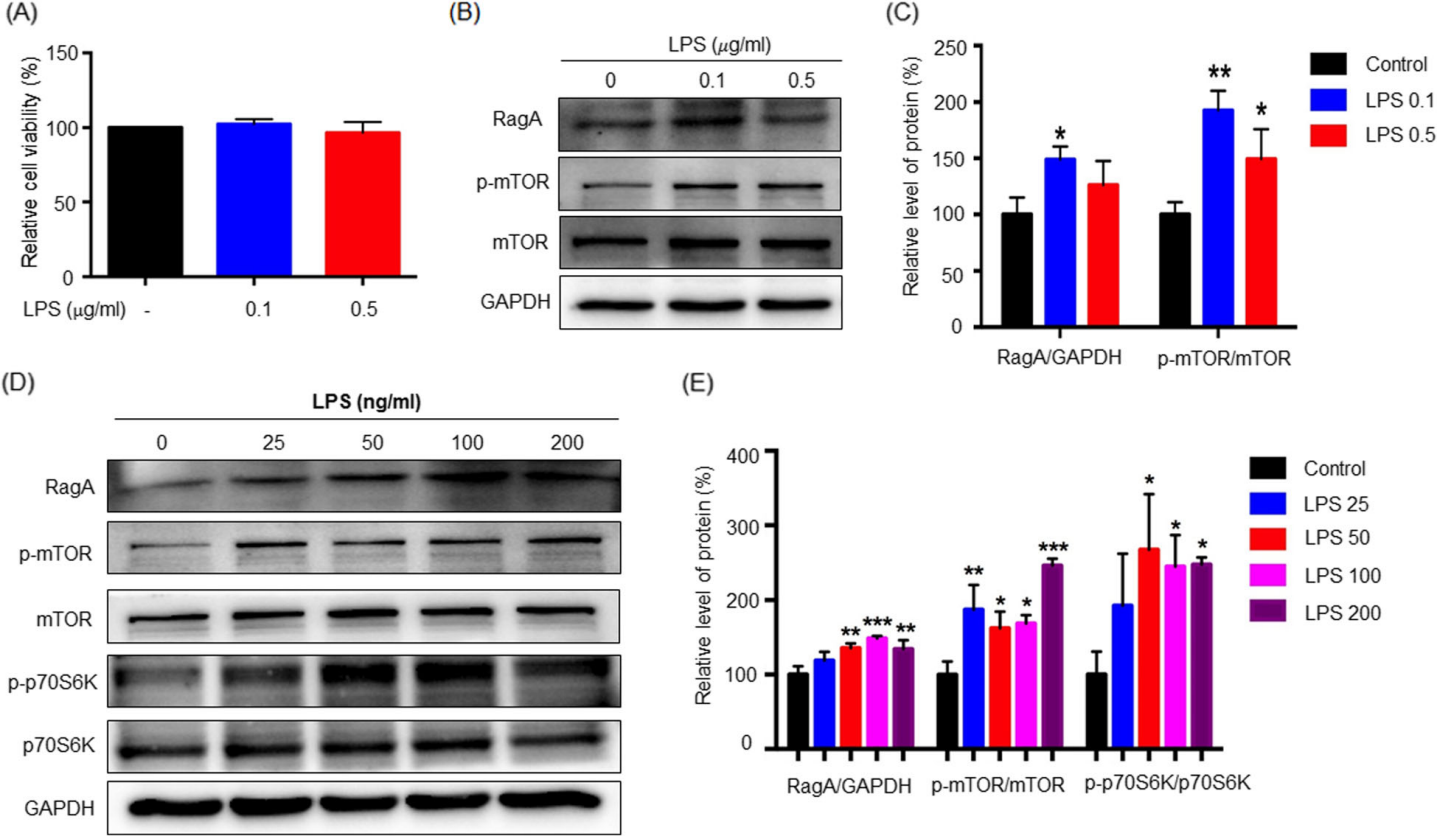
LPS induced RagA expression and mTOR-p70S6K activation in the primary cortical neurons and neuronal stem cells. **a** Optimization of LPS concentration. After 24 h LPS treatment, primary rat cortical neurons were examined by staining with Hoechst 33342 and PI. PI-positive and Hoechst-stained cells were enumerated under a Zeiss fluorescence microscope (Carl Zeiss, Jena, Germany) and analyzed by one-way ANOVA followed by Tukey’s test (n = 3). *p < 0.05. **b** Activation of RagA and mTOR pathways in LPS-stimulated primary neurons. After 24 h LPS treatment, primary cortical neurons were lysed and analyzed by western blotting for RagA, mTOR, phospho-mTOR expression, while GAPDH was analyzed as control. Representative blot was shown. **c** Quantitative analysis of RagA and p-mTOR. Western blots in **b** were determined by a densitometric method. The signals (means ± SD) from three independent experiments were analyzed by one-way ANOVA, followed by post hoc Tukey’s test. *p < 0.05; **p < 0.01 (LPS vs control). **d** Activation of RagA and mTOR pathways in LPS-stimulated neuronal stem cells. After 24 h LPS treatment, neuronal stem cells were lysed and analyzed by western blotting for the expression of RagA, mTOR, phospho-mTOR, p70S6K, and phospho-p70S6K, while GAPDH was analyzed as control. Representative blot was shown. **e** Quantitative analysis of RagA, phospho-mTOR, and phospho-p70S6K. Western blots in **d** were determined by a densitometric method. The signals (means ± SD) from three independent experiments were analyzed by one-way ANOVA, followed by post hoc Tukey’s test. *p < 0.05; **p < 0.01; ***p < 0.001 (LPS vs control)

To confirm the effect of LPS on the expression of RagA and p-mTOR, we firstly determined LPS concentrations for the treatment of primary cortical neurons [37, 38]. After 24 h treatment with 0.1 or 0.5 μg/ml of LPS, the primary cortical neurons did not show much positive staining by necrotic indicator PI (Fig. 3a). The cellular proteins were subsequently extracted from the primary cortical neurons for western blot analysis with specific antibodies. As shown in Fig. 3b–c, LPS (0.1 μg/ml) effectively induced the expression of RagA and p-mTOR in the primary cortical neurons (*p* < 0.05 and *p* < 0.01, respectively).

### LPS induced the activation of mTORC1 in the neural stem cells

The effects of LPS on the RagA, mTOR, and p70S6K signaling pathways were further examined in the neural stem cells [31]. After 24 h LPS treatment, the cellular proteins were extracted and analyzed by western blotting. As shown in Fig. 3d, e, LPS profoundly induced the expression of RagA and activation of p-mTOR in the neural stem cells (*p* < 0.001) and also increased the expression of p-p70S6K to a certain extent (*p* < 0.05).

### LPS induced the lysosomal accumulation of mTORC1 in the neural stem cells

**Fig. 4:**
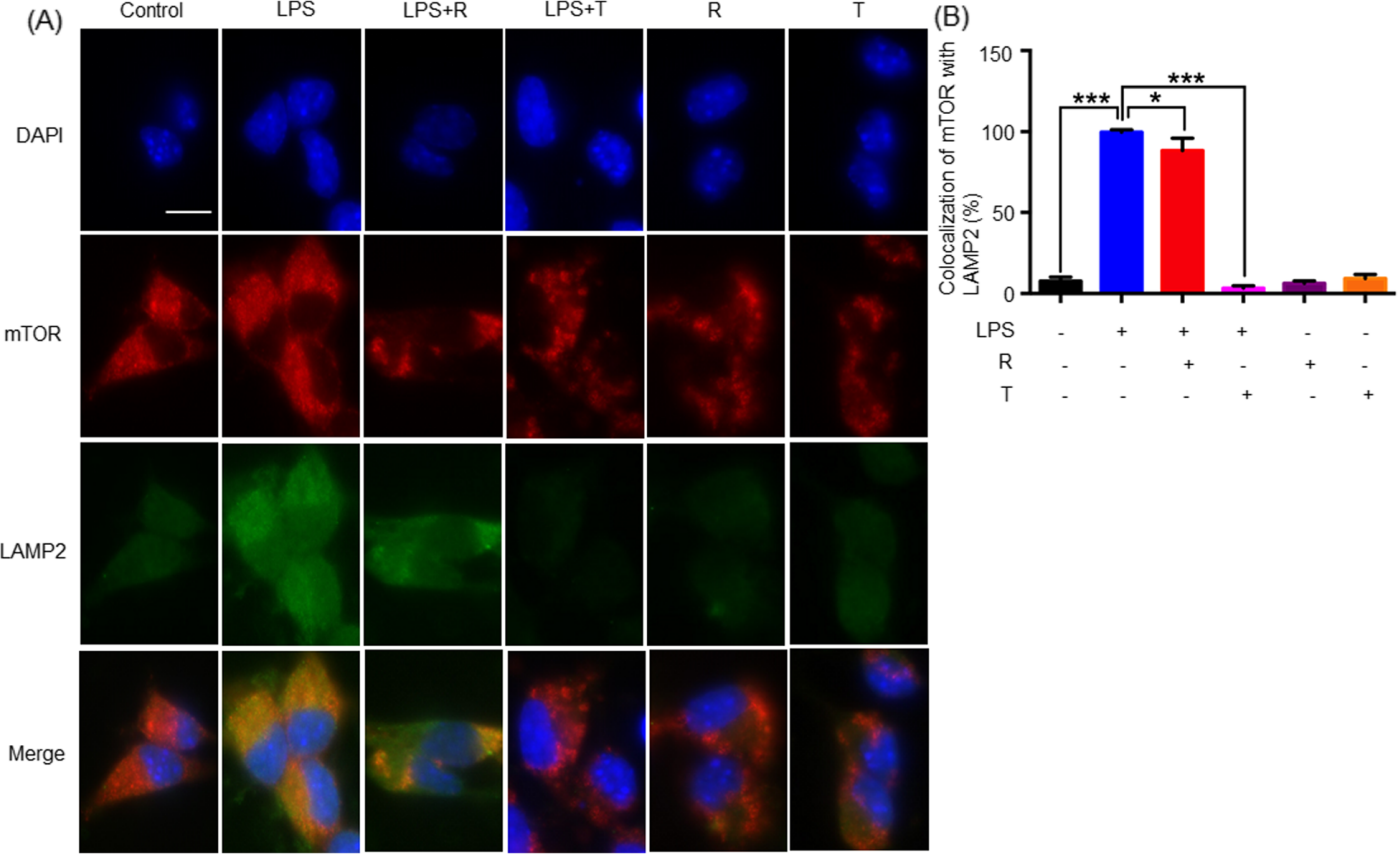
Immunofluorescence staining of LPS-induced mTORC1 translocation to the lysosomal membrane. **a** Neural stem cells were treated with 200 ng/ml of LPS, 20 nM Rapamycin (R), and 1 μM Temsirolimus (T) for 24 h. Following the treatment, the cells were stained with specific antibodies against mTOR and LAMP2, while the cell nuclei were indicated by DAPI staining. The stained cells were imaged on a Zeiss fluorescence microscope (Carl Zeiss, Jena, Germany). **b** Quantitative analysis of mTOR and LAMP2 levels. Co-localization of mTOR and LAMP2 signals in the neuronal stem cells was quantified by a densitometric method and expressed in mean ± SD (*n* = 3). The difference between the experimental groups was analyzed by one-way ANOVA, followed by post hoc Tukey’s test. *p < 0.05; ***p < 0.001 (LPS vs control/LPS + R/LPS + T)

To clarify the effects of LPS on the activation of mTORC1, we treated neural stem cells with LPS (200 ng/ml), mTORC1 inhibitor Temsirolimus (1 μM), and mTOR inhibitor Rapamycin (20 nM), alone or in combination, for 24 h. As shown in Fig. 4a, b, LPS not only markedly increased the expression levels of lysosomal biomarker LAMP2, but also effectively induced the co-localization of mTOR with LAMP2. Both Temsirolimus and Rapamycin downregulated the expression of mTOR. Interestingly, Temsirolimus strongly inhibited the translocation of mTORC1 to the lysosomal surface whereas Rapamycin showed a slight effect.

### LPS induced the activation of mTORC1 in the PC12 cells via upregulating RagA

**Fig. 5:**
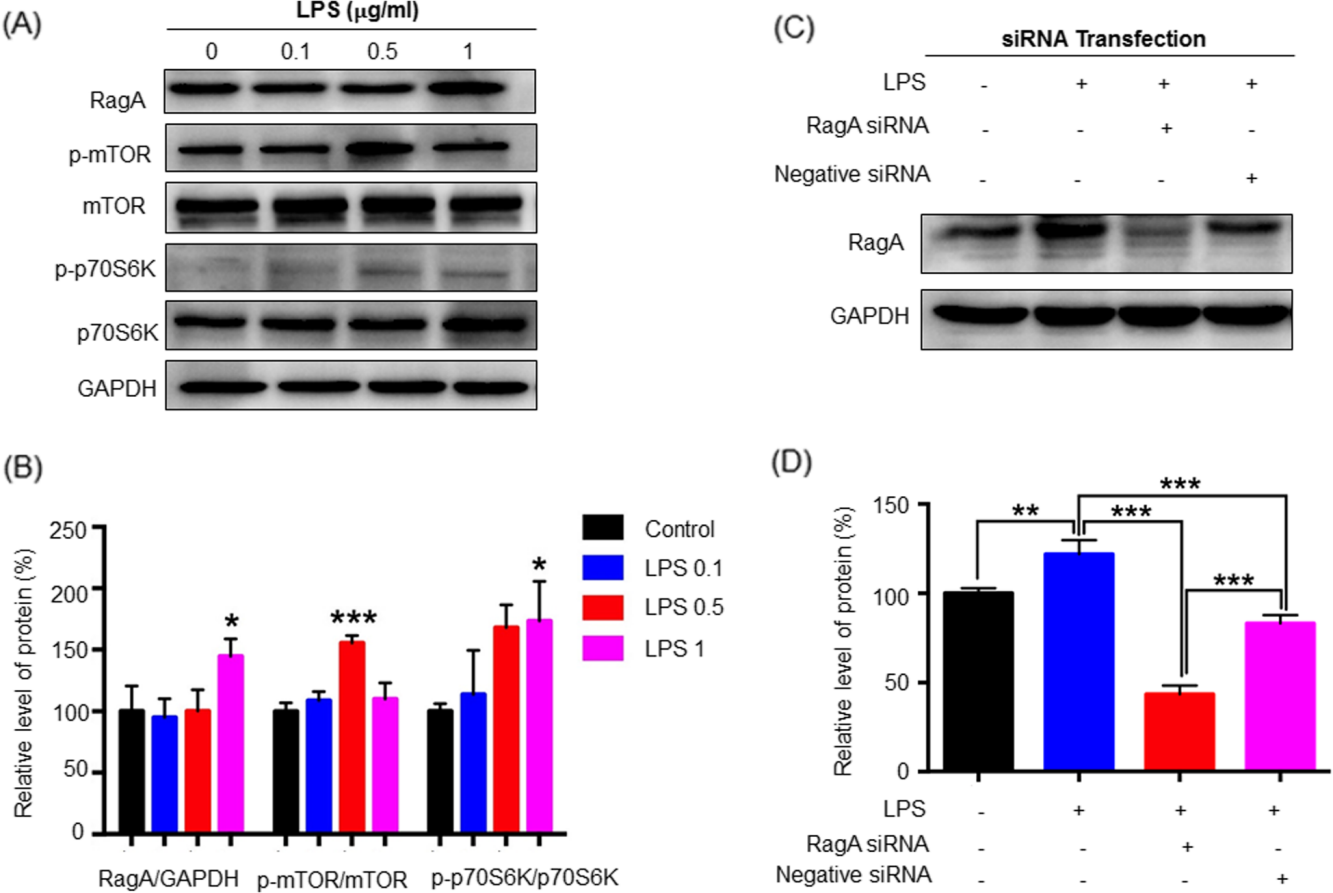
Examination of RagA overproduction in the activation of mTOR-p70S6K pathway in LPS-treated PC12 cells. **a** LPS induced the activation of RagA, mTOR, and p70S6K. After 24 h drug treatment, PC12 cells were lysed and analyzed by western blotting with antibodies against RagA, mTOR, phospho-mTOR, p70S6K, and phospho-p70S6K, whereas GAPDH was analyzed as control. Representative blot was shown. **b** Quantitative analysis of protein expression. Western blots in **a** were determined by a densitometric method. The signals (means ± SD) from three independent experiments were analyzed by one-way ANOVA, followed by post hoc Tukey’s test. *p < 0.05; **p < 0.01; ***p < 0.001 (LPS vs control). **c** Western blotting analysis for siRNA-mediated RagA silencing. PC12 cells were transfected with RagA-specific and negative control siRNAs for 72 h. The transfected cells were treated with 1 μg/ml of LPS for 24 h. The cells were analyzed by western blotting for RagA expression, whereas GAPDH was analyzed as control. Representative blot was shown. **d** Quantitative analysis of RagA silencing efficiency. Western blots in **c** were determined by a densitometric method. The signals (means ± SD) from three independent experiments were analyzed by one-way ANOVA, followed by post hoc Tukey’s test. *p < 0.05; **p < 0.01; ***p < 0.001 (LPS vs control/ LPS + RagA siRNA/ LPS + negative siRNA)

**Fig. 6:**
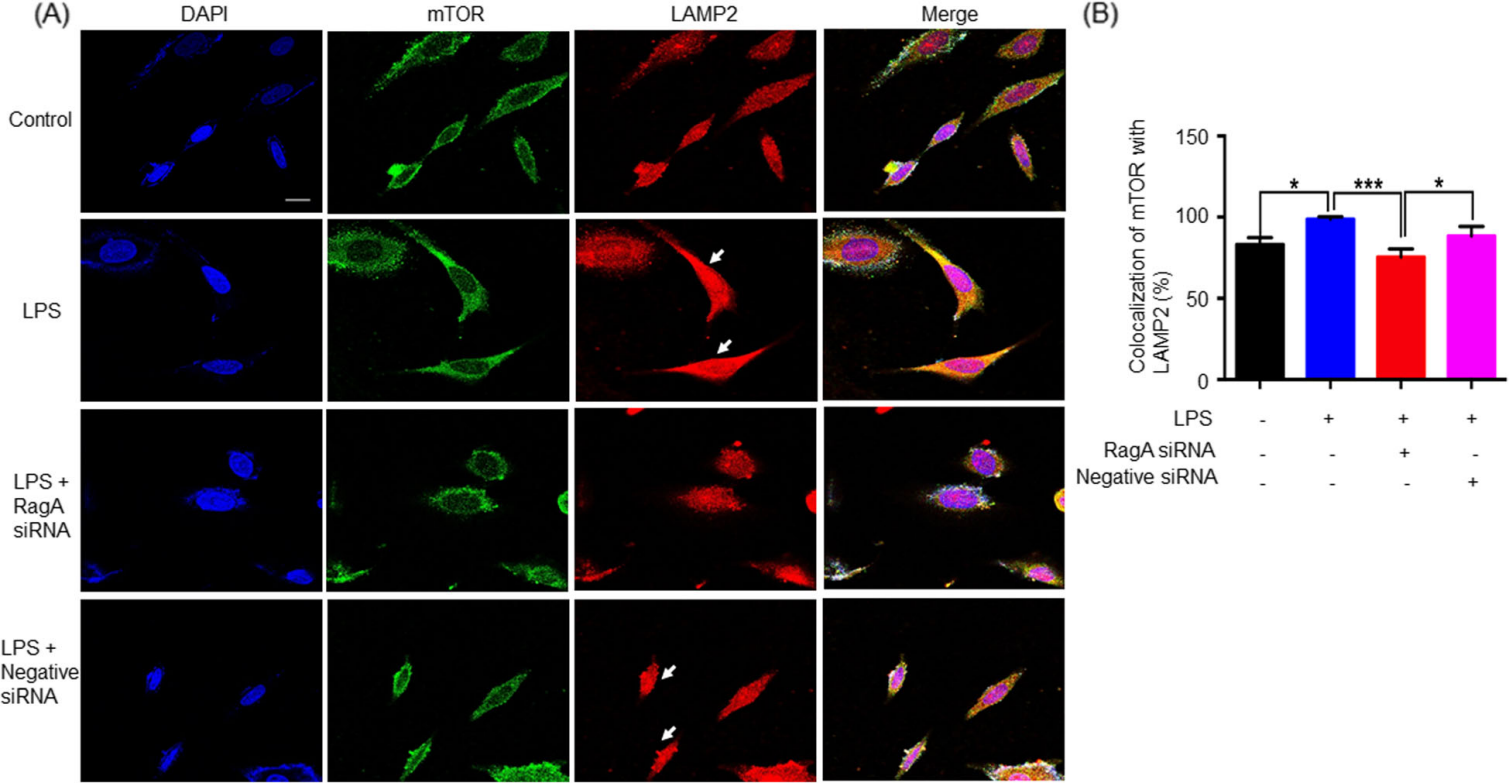
RagA silencing blocked LPS-induced translocation of mTORC1 towards lysosomal membrane. **a** After siRNA transfection, PC12 cells were treated with 1 μg/ml of LPS for 24 h, and stained with specific antibodies against mTOR and LAMP2, while the cell nuclei were indicated by DAPI staining. The stained cells were imaged on a Zeiss LSM 700 confocal fluorescence microscope (Carl Zeiss, Jena, Germany). **b** Quantitative analysis of mTOR and LAMP2 levels. Co-localization of mTOR and LAMP2 signals in the PC12 cells was quantified by a densitometric method and expressed in mean ± SD (*n* = 3). The difference between the experimental groups was analyzed by one-way ANOVA, followed by post hoc Tukey’s test. *p < 0.05; **p < 0.01; ***p < 0.001 (LPS vs control/ LPS + RagA siRNA or LPS + RagA siRNA vs LPS + negative siRNA)

To determine whether RagA plays a role in LPS-induced activation of mTORC1, we silenced RagA expression by introducing RagA-specific siRNA into PC12 cells. In practical, we firstly validated the effects of LPS on the expression of RagA, p-mTOR, and p-p70S6K in PC12 cells. As shown in Fig. 5a, b, LPS (1 μg/ml or 0.5 μg/ml) effectively upregulated the expression of RagA and increased the levels of p-mTOR and p-p70S6K in PC12 cells [39]. Secondly, we verified the siRNA-mediated silencing of RagA expression in PC12 cells. We transfected PC12 cells with RagA siRNA or negative control siRNA prior to LPS challenge. As a result, RagA siRNA transfection effectively reduced the level of RagA protein in response to LPS, while negative control siRNA transfection altered the expression level of RagA protein against LPS challenge (Fig. 5c, d). Thirdly, we examined the lysosomal translocation of mTOR in RagA siRNA transfected cells. As shown in Fig. 6a, b, LPS induced the co-localization of mTORC1 with lysosomal biomarker LAMP2, indicating the translocation of mTORC1 to the lysosomal membranes. RagA siRNA transfection inhibited LPS-induced translocation of mTORC1 to the lysosomal membranes compared with the activities of negative control siRNA.

## Discussion

Bacterial infection triggers inflammation in depression, bipolar disorder, and other mental health problems [40]. The neutrophil-to-lymphocyte ratio is clinically determined to index inflammation in the early onset of depression and monitor the suicidal vulnerability in patients [41]. Nevertheless, the existing antidepressant drugs may exacerbate the inflammatory responses in the brain [42]. The objective of this study was to explore the link between bacterial infection and depression. We focused on the role of small GTPase RagA in the activation of mTOR-p70S6K pathway towards depressive-like behaviors.

Neuroinflammation changes the expression profiles of proinflammatory cytokines, which promotes the migration of monocytes to the central nervous system and cause the depressive and anxiety-like behaviors [43]. Bacterial LPS is well-known to induce neuroinflammation in rat brain [44]. LPS was recently shown to induce depressive-like behaviors in mice via activating the NLRP3 inflammasome [45]. LPS stimulates the release of several inflammatory factors (e.g., TNF-α, IL-1β, and IL-6), and worsens depressive-like behaviors in mice suffering from unpredictable chronic mild stress [19]. These studies consolidate the potential role of endotoxic stress in depression. Interestingly, many scientists used endotoxin LPS to induce animal models of inflammation and depression for preclinical studies [46]. The present study was designed to examine the importance of RagA in endotoxic stress. We firstly validated that LPS induced depressive-like behaviors in C57BL/6 N mice by assessing depressive behaviors with FST and TST (Fig. 1b, c).

Four Rag GTPase isoforms form heterodimers (e.g., RagA/B and RagC/D) to sense the availability of different amino acids such as serine and glutamine [20, 22]. When the amino acid is deficient, Rag heterodimer interacts with the trimeric tuberous sclerosis complex (TSC) at the lysosomal surface and inactivates Ras-homolog enriched in the brain (Rheb). SH3BP4 prevents the interaction between Rag heterodimer and mTORC1. When the amino acid is deprived, thus, mTORC1 is inactive. On the other hand, upon activation by amino acids, active Rag heterodimer (RagA/B^GTP^ and RagC/D ^GDP^) promotes the translocation of mTORC1 to the lysosomal surface where Rheb resides [22]. mTORC1 activation further induces the phosphorylation of p70S6K [20, 47]. RagA appeared to be important to activate mTORC1 for autophagy and cell survival [48]. mTORC1 signal is transduced by two downstream targets, p70S6K and 4EBP1, respectively. p70S6K and 4EBP1 are implicated in the control of protein synthesis, cell survival, and proliferation, as well as in the development of behavioral sensitization to psychological stimuli including methamphetamine [49, 50]. p70S6K might be a target in the regulation of depressive behaviors in SD rats [27]. Interestingly, ketamine exhibits rapid antidepressant activity through the activation of mTOR signaling pathway [51]. In the present study, we analyzed the expression of RagA, mTOR, and p70S6K in mouse brains following the LPS challenge. As a result, LPS not only increased the expression of RagA, but also induced the phosphorylation of mTOR and p70S6K. These results suggested a link between RagA and mTOR signaling pathway in LPS stimulation. We further confirmed that RagA was co-localized with neuron marker NeuN in the forebrain cortex from LPS-treated mice by immunofluorescence staining. These results suggested that LPS stimulation could upregulate the expression of small GTPase RagA and induce the activation of mTOR and p70S6K in mouse neurons.

It was recently found that the adult neurogenesis in the hippocampus was impaired before the symptoms of depression episode appeared [52]. In the present study, we further verified the correlation between the expression levels of RagA and the activation of mTOR and p70S6K in primary neurons, neuronal stem cells, and PC12 cells. Firstly, when primary cortical neurons were treated with LPS at the sub-toxic concentrations. The expression levels of RagA and p-mTOR were positively correlated. Secondly, upon LPS stimulation, neural stem cells expressed more RagA and showed higher levels of p-mTOR and p-p70S6K. In addition, LPS stimulation not only upregulated the expression of the lysosomal biomarker LAMP2 but also induced the translocation of mTORC1 to the lysosomal membranes. These results suggest a cascade for RagA upregulation, mTORC1 lysosomal translocation, mTOR activation, and p70S6K activation. Thirdly, following siRNA-mediated silencing of RagA expression, PC12 cells failed to respond to LPS and no longer allowed the activation of mTORC1. Collectively, these results suggested that LPS might induce the activation of mTOR and p70S6K via upregulating RagA expression.

## Conclusions

**Fig. 7:**
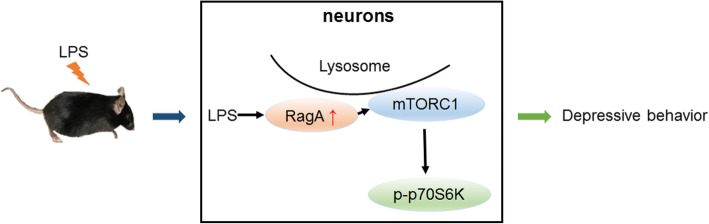
A diagram of the proposed mechanism on LPS-induced depressive-like behaviors in mice

The present study for the first time demonstrated LPS could activate mTOR pathway via upregulating RagA expression. Thus, the sequential activation of RagA, mTOR, and p70S6K could be a potential mechanistic link between bacterial infection and depression (Fig. 7). We propose that small GTPase RagA may be a potential therapeutic target for the development of anti-depressant drugs.

## Data Availability

The data are available from the corresponding author on a reasonable request.

## Abbreviations

4EBP1: Eukaryotic translation initiation factor 4E-binding protein 1
DAPI: 4′-6-diamidino-2- phenylindole
DMEM: Dulbecco’s modified Eagle’s medium
E14: Embryonic day 14
ECL: Select chemiluminescence
FBS: Fetal bovine serum
FST: Forced swim test
HKU*-*SIRI: The University of Hong Kong Shenzhen Institute of Research and Innovation
HS: Horse serum
IP: Intraperitoneal
LAMP2: Lysosome-associated membrane protein 2
LPS: Liposaccharide
mTOR: Mammalian target of rapamycin
mTORC1: mTOR complex 1
mTORC2: mTOR complex 2
p70S6K: p70 ribosomal S6 kinase
PBS: Phosphate-buffered saline
PI: Propidium iodide
Rheb: Ras-homolog enriched in brain
SD: Sprague–Dawley
TBS-T: Tris-buffered saline containing 0.1% Tween-20
TSC: Trimeric tuberous sclerosis complex.
TST: Tail suspension test
